# Mechanisms linking parental educational attainment with child ADHD, depression, and academic problems: a study of extended families in The Norwegian Mother, Father and Child Cohort Study

**DOI:** 10.1111/jcpp.13197

**Published:** 2020-01-19

**Authors:** Fartein Ask Torvik, Espen Moen Eilertsen, Tom A. McAdams, Kristin Gustavson, Henrik Daae Zachrisson, Ragnhild Brandlistuen, Line C. Gjerde, Alexandra Havdahl, Camilla Stoltenberg, Helga Ask, Eivind Ystrom

**Affiliations:** ^1^ Centre for Fertility and Health Norwegian Institute of Public Health Oslo Norway; ^2^ Department of Psychology University of Oslo Oslo Norway; ^3^ Norwegian Institute of Public Health Oslo Norway; ^4^ Social, Genetic & Developmental Psychiatry Centre Institute of Psychiatry, Psychology & Neuroscience King's College London London UK; ^5^ Promenta Research Centre University of Oslo Oslo Norway; ^6^ Department of Special Needs Education University of Oslo Oslo Norway; ^7^ Nic Waals Institute Lovisenberg Diaconal Hospital Oslo Norway; ^8^ MRC Integrative Epidemiology Unit University of Bristol Bristol UK; ^9^ Department of Global Public Health and Primary Care University of Bergen Bergen Norway; ^10^ PharmacoEpidemiology and Drug Safety Research Group School of Pharmacy University of Oslo Oslo Norway

**Keywords:** Attention deficit hyperactivity disorder, depression, academic problems, educational attainment, MoBa

## Abstract

**Background:**

Low educational attainment in parents is associated with child psychopathology. It is not clear whether the associations are due to risk factors that family members share or due to effects of maternal or paternal education on the offspring. We investigate whether associations between maternal and paternal educational attainment and child symptoms of attention deficit/hyperactivity disorder (ADHD), depression, and academic problems are due to shared genetic factors, shared family environmental factors, or effects of the parental phenotype educational attainment itself.

**Methods:**

This study is based on the Norwegian Mother, Father and Child Cohort Study (MoBa). The sample comprised 34,958 children (17,128 girls) in 28,372 extended‐family units. We used data from related nuclear families linked by siblings in the parent generation. We applied a quasi‐experimental extended children‐of‐twins design that included siblings in both generations and took account of nonrandom mating by including partners. Educational attainment was self‐reported by mothers and fathers. Mothers reported children's symptoms of ADHD, symptoms of depression, and academic problems by questionnaire when the children were 8 years old.

**Results:**

Children of lowly educated parents scored higher on all outcomes and had an approximate doubling of the risk of high symptom levels. The association between maternal and paternal educational attainment and child symptoms of ADHD and academic problems persisted after controlling for shared genetic and family environmental factors. Phenotypic transmission to depression was weaker and in the best fitting model fully explained by genetic factors shared by the two generations.

**Conclusions:**

Associations between educational attainment of mothers and fathers and child symptoms of ADHD and academic problems could not be ascribed to shared familial risk factors, whereas associations with symptoms of depression could. Parental education or resources and behaviors resulting from low education might be targets of interventions aimed at reducing symptoms of ADHD and academic problems.

## Introduction

Children of parents with low education have a twofold to threefold increased risk of psychiatric disorders such as attention deficit hyperactivity disorder (ADHD) and depression (Hjern, Weitoft, & Lindblad, 2010; Russell, Ford, Williams, & Russell, 2016), compared to children of parents with high education. They also have more academic problems (Sirin, 2005), which is strongly related to mental health (McLeod, Uemura, & Rohrman, 2012). Understanding why these intergenerational associations exist may provide opportunities to improve outcomes for these children. One or more of several possible mechanisms may underlie these associations.

First, quantitative and molecular genetic studies have shown genetic correlations between low educational attainment and ADHD (.54) and depression (.15) (Demontis et al., 2019; Howard et al., 2019). When these genetic variants are transmitted to the next generation, an association between low parental education and child outcomes can arise even in the absence of direct effects (i.e. pleiotropy). Pleiotropic effects thus constitute a *confounding* of the parent–offspring association. Substantiating this, polygenic scores for high educational attainment in adults predict fewer symptoms of ADHD (de Zeeuw et al., 2014), fewer internalizing symptoms, and better school performance (Jansen et al., 2018) in children. Quantitative and molecular genetic studies account for different amounts of variance, but both kinds of studies indicate that genetic transmission could partially explain parent–child associations.

Second, parents and children share social environments that can confound the parent–offspring associations, such as the neighborhood they live in (Chetty, Friedman, Hendren, Jones, & Porter, 2018). Environmental factors shared in families are important for child psychopathology (Bergen, Gardner, & Kendler, 2007) and for educational attainment (Branigan, McCallum, & Freese, 2013). It is unknown to what degree the social environments that influence parental education also influence offspring psychopathology and academic problems.

Third, the parental educational attainment may be associated with child outcomes over and above the genetic and environmental risk factors that families share across generations. Low education could influence material conditions, parenting skills, social development, or stress, and thereby influence children's mental health and learning (Bradley & Corwyn, 2002; Solis et al., 2015). For instance, an adoption study showed that rearing environment influences depression (Kendler, Ohlsson, Sundquist, & Sundquist, 2018). Quasi‐experimental studies have indicated that parental education plausibly exerts some influences on the education of adult offspring (Black, Devereux, & Salvanes, 2005; Kong et al., 2018), although the effect of parental education on academic problems in childhood is unknown. Few studies have examined the mode of transmission from parental educational attainment to child psychopathology, but low income could partly reflect the same socioeconomic adversity as low education. In a natural experiment where Native Americans received positive income shocks, there was a reduction in behavior problems (Costello, Compton, Keeler, & Angold, 2003) and emotional symptoms (Akee, Copeland, Costello, & Simeonova, 2018) among their children. This implies that *causal influences* are possible, but we do not know if the effect of education will mirror that of income, and whether such effects can explain social differences in societies with low rates of absolute deprivation. Causal influences are a possible explanation of the observed genetic associations (Turkheimer, Pettersson, & Horn, 2014), because genetic factors can influence parenting style (Wertz et al., 2019). Most studies are not able to separate between causal and noncausal modes of intergenerational transmission.

Behavior genetic studies such as the children‐of‐twins design can distinguish between the different mechanisms of intergenerational association (McAdams et al., 2018). This quasi‐experimental approach is useful when it is unfeasible to assign children to experimental conditions (Pingault et al., 2018). The phenotypic effect is estimated as the remaining association after accounting for genetic transmission and shared environments. A limitation of most studies of this kind is that they have only included data on one of the parents, included as part of a twin or sibling pair, and assume that there is no partner resemblance. However, partners strongly resemble each other on educational attainment (Greenwood, Guner, Kocharkov, & Santos, 2014). Excluding a parent can bias the results and lead to false identification of phenotypic effects. We applied an extended children‐of‐twins‐and‐siblings model that included partners and multiple offspring per sibling to investigate genetic, shared environmental, and phenotypic transmission underlying associations between maternal and paternal educational attainment and symptoms of ADHD, symptoms of depression, and academic problems in school‐aged children.

## Methods

### Sample

The Norwegian Mother, Father and Child Cohort Study (MoBa) is a prospective population‐based pregnancy cohort study conducted by the Norwegian Institute of Public Health (Magnus et al., 2016). Participants were recruited from all over Norway from 1999 to 2008. The women consented to participation in 40.6% of the eligible pregnancies. The cohort includes 114,500 children, 95,200 mothers, and 75,200 fathers. The current study is based on version 11 of the quality‐assured data files released for research in 2018. In the present study, we use data on children whose mothers and fathers reported educational attainment in pregnancy. Mothers reported psychosocial outcome variables when the children were 8 years old. The design was based on comparing members of extended‐family units, which included pairs of siblings, their partners, and their children (i.e. two related nuclear families). The family structure of the MoBa participants was mapped through a link with the population register at Statistics Norway. For families with multiple children, we selected two children at random. A unit thus included up to eight individuals: two siblings in the parent generation, two partners, and four offspring. The resulting sample comprised a total of 34,958 children (49.0% girls) in 28,372 extended‐family units. Among these were 112 children of monozygotic (MZ) twins, 97 children of dizygotic (DZ) twins, 11,867 children of full siblings, 349 children of maternal half‐siblings, and 414 children of paternal half‐siblings. In the parent generation, we had valid data on 72,582 mothers and 70,107 fathers, among which there were 191 MZs, 218 DZs, 25,061 full siblings, 778 maternal half‐siblings, and 909 paternal half‐siblings.

### Ethics

MoBa has been approved by the *Regional Committees for Medical and Health Research Ethics* and the *Norwegian Data Inspectorate*. The present study has a separate approval (2013/863).

### Measures

#### Parental educational attainment

Educational attainment was assessed in the questionnaires given to mothers and fathers at pregnancy. Six response categories were available, corresponding to 1 = ‘Primary and lower secondary school’ (9 years; mandatory education), 2 = ‘Upper secondary school, basic (1–2 years’ (10–11 years), 3 = ‘Vocational upper secondary school’ (11 years), 4 = ‘Upper secondary school, completed’ (12 years), 5 = ‘First stage of higher education, undergraduate level’ (15 years), and 6 = ‘Second stage of higher education, graduate level’ (17 years). Level of education (1–6) was used as the unit on these variables.

#### Symptoms of ADHD

Mothers reported symptoms of ADHD on the ADHD index of the Parent/Teacher Rating Scale for Disruptive Behavior Disorders (Silva et al., 2005). The scale consists of 18 items describing the child's behavior during the last six months and reflects symptoms of ADHD from the Diagnostic and Statistical Manual of Diseases – 4th revision (DSM‐IV). Items were scored along four ordered categories. Responses were summed (α = .91) and scaled to T‐scores (i.e. mean = 50; *SD* = 10).

#### Symptoms of depression

Mothers reported symptoms of offspring depression on the Short Mood and Feelings Questionnaire (Angold et al., 1995). The scale consists of 13 descriptive phrases regarding how the child had been feeling or acting during the last 2 weeks. Each item had three ordered response options. Items were summed (α = .78) and T‐scaled.

#### Academic problems

All Norwegian children take mandatory screening tests in reading and arithmetic skills. Teachers usually inform parents of the results, and mothers were asked to report the results of the reading and arithmetic tests by four response options: ‘1 – Master subject well’, ‘2 – Must work more but teacher is not concerned’, ‘3 – Teacher is concerned’, and ‘4 – Don't know/not discussed with teacher’. Responses with the value 4 were coded as missing. The items were summed (α = .78) and T‐scaled.

### Statistical analyses

Associations across generations were first investigated using linear regression and continuous scores. In order to describe the associations on a metric that is comparable to diagnostic studies, we also dichotomized the symptoms of ADHD and symptoms of depression at the 95th percentile and defined children who did not master reading nor arithmetic well as having academic problems (13%).

**Figure 1:**
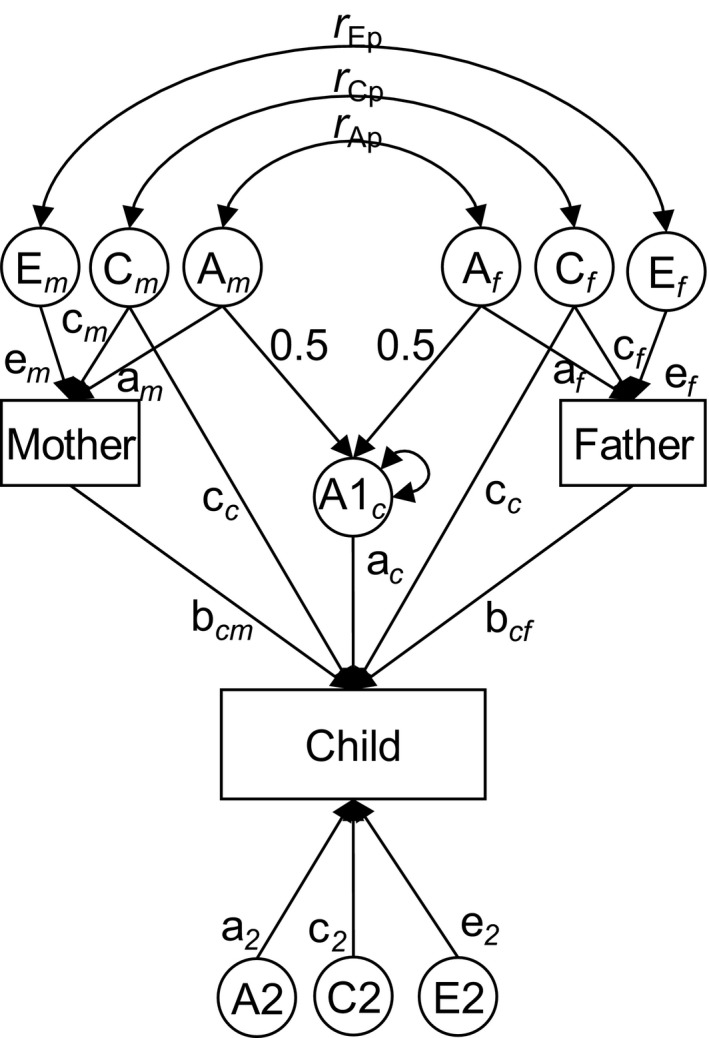
Partial path diagram of the extended children‐of‐twins model. The illustration shows only one parent–child trio, whereas the model fitting included twins and siblings in the parent generation and up to two children per nuclear family. A = additive genetic factors; C = shared (family) environmental factor; E = individual‐specific environmental factors

Mechanisms of intergenerational transmission were then investigated with biometric structural equation models known as children‐of‐twin models (Keller et al., 2009; McAdams et al., 2018; Silberg, Maes, & Eaves, 2010). In these analyses, we used the continuous scores scaled as described above (level of education and T‐scores). T‐scores can be converted to Z‐scores by subtracting 50 and dividing by 10. The models use the genetic relatedness between different classes of relatives to distinguish various modes of intergenerational transmission. For instance, if a child of an MZ twin is more similar to her father than to the genetically identical uncle, this indicates an effect of the parental phenotype. This provides a counterfactual condition that can be used to answer the question: ‘What would happen if two persons had genetically identical parents, but grew up in different families?’. In addition to twins, our design included parental full siblings and half‐siblings, and up to two full‐sibling offspring per family, which increases the statistical power of the design (McAdams et al., 2018). A partial path diagram illustrating key concepts of the model is shown in Figure 1 for a mother–father pair with one child. The full model is shown in Figure S1. Under an additive genetic model, the genetic correlation is assumed to be 1 between MZ twins, 1/2 between DZ twins and full siblings, and 1/4 between half‐siblings. The respective genetic correlations between their offspring are 1/4, 1/8, and 1/16. These differences can be used to divide variation in traits into additive genetic (A), shared environmental (C), and individual‐specific environmental (E) components. C can be substituted for dominant genetic effects (D). The availability of data on both parents and on in‐laws allowed us to estimate partner correlations between these components (*r*_Ap_, *r*_Cp_, and *r*_Ep_) and to correct for nonrandom mating. For instance, an individual‐specific partner correlation (*r*_Ep_) above zero could have been misidentified as a phenotypic effect of the included parent if not modeled. The genetic partner correlation (*r*_Ap_) is used to adjust the genetic sibling correlation to above .50, which is necessary to correctly distinguish between additive genetic and shared environmental factors.

Associations between parents and children were decomposed into three modes of intergenerational association: passive genetic transmission (*a*_c_), which is the effect that genetic influences on parental educational attainment have on the child outcomes; passive shared environmental transmission (*c*_c_), which is the effect that shared environmental influences on parental educational attainment have on the offspring outcome; and phenotypic effects of mother (*b*_cm_) and fathers (*b*_cf_), which are the effects of maternal and paternal educational attainment, respectively, on the offspring outcome after accounting for intergenerationally shared risk factors (*a*_c_ and *c*_c_). If a factor influences the education of an individual, but not the sibling's education, and this factor is associated with an outcome in the individual's offspring, but not in the sibling's offspring, that could indicate effects of the educational attainment on the offspring outcome. Such factors could include individual experiences, which are included in E, and genetic factors that were not transmitted to the next generation (included in A, but not in A1). Residual variation in offspring outcomes that was independent of parental educational attainment was decomposed into separate Aʹ, Eʹ, and either Cʹ or Dʹ components. For each outcome, we compared models that included any combination of the three modes of intergenerational transmission. We compared the fit of the alternative models using the Akaike information criterion (AIC). Models with low AIC are preferred because they provide a better balance between complexity and fit to the data (Akaike, 1987). The models were fitted using OpenMx.

## Results

Mothers were on average 30.2 (*SD* = 4.5) and fathers 32.6 years old (*SD* = 5.4) when recruited. The educational level of mothers and fathers is shown in Table 1 along with the average scores on symptoms of ADHD, symptoms of depression, and academic problems of children in each educational group. Children of parents with high educational attainment scored lower on all outcomes compared to children of parents with low educational attainment. The correlation for educational attainment between mothers and fathers was 0.49 (95% CI 0.49, 0.50). Sibling discordance in educational attainment for half‐siblings, full siblings, and twins in the parental generation is detailed in Table S1. Table S2 shows the phenotypic correlations between parental educational attainment and child outcomes. Figure S2 shows the distributions of the child outcomes before and after logarithmic transformation.

**Table 1:** Average level of symptoms of attention deficit hyperactivity disorder (ADHD), symptoms of depression, and academic problems by sex and parental educational attainment

Variable/level	*N*	%	ADHD		Depression		Academic problems	
			Mean	*SD*	Mean	*SD*	Mean	*SD*
Sex								
Boys	17,830	51.0	51.7	10.7	50.2	10.2	50.4	10.2
Girls	17,128	49.0	48.2	8.8	49.8	9.8	49.6	9.8
Maternal education								
1. Lower secondary school (9 years)	423	1.3	54.1	12.7	54.1	13.7	52.8	12.4
2. Upper secondary, basic (10.5 years)	1,017	3.1	51.8	11.3	51.6	12.1	51.9	11.0
3. Vocational (11 years)	3,426	10.3	51.6	11.4	51.0	10.9	52.2	11.6
4. Upper secondary, completed (12 years)	4,042	12.2	50.8	10.8	50.8	10.8	51.0	10.6
5. University, short (15 years)	15,080	45.5	49.3	9.4	49.5	9.4	49.7	9.6
6. University, long (17 years)	9,123	27.6	49.6	9.4	49.5	9.4	48.9	9.2
Paternal education								
1. Lower secondary school (9 years)	1,014	3.1	52.7	12.4	52.3	11.9	52.5	12.1
2. Upper secondary, basic (10.5 years)	1,749	5.4	51.6	11.5	51.3	11.4	51.6	11.2
3. Vocational (11 years)	7,757	23.9	50.7	10.8	50.6	10.6	51.4	10.9
4. Upper secondary, completed (12 years)	3,800	11.7	50.0	9.8	50.1	10.0	49.7	9.7
5. University, short (15 years)	9,382	28.9	49.4	9.3	49.5	9.5	49.5	9.4
6. University, long (17 years)	8,716	26.9	49.3	9.2	49.3	9.3	48.7	9.0

*N*, number of observations; *SD*, standard deviation. All outcomes are T‐scaled with a mean of 50 and standard deviation (*SD*) of 10. Numbers in parenthesis correspond to the typical total length of education.

Table S3 shows linear regression estimates of the association between parental educational attainment and the child outcomes adjusted for sex and age. Girls had fewer problems than boys on all outcomes. The proportions of children with high scores on ADHD, depression (above 95th percentile), or academic problems (did not master reading nor arithmetic well) by maternal and paternal educational attainment are shown in Table 2 along with the number of observations in each category. Compared to children of two parents with the longest education, children of two parents with the shortest education were 4.46 (95% CI 2.64, 7.54) times more likely to have high levels of ADHD, 3.27 (95% CI 1.77, 6.08) times more likely to have high levels of depression, and 3.39 (95% CI 2.29, 5.03) times more likely to have academic problems. Results of binary logistic regression using the dichotomous outcomes are shown in Table S4.

**Table 2:**
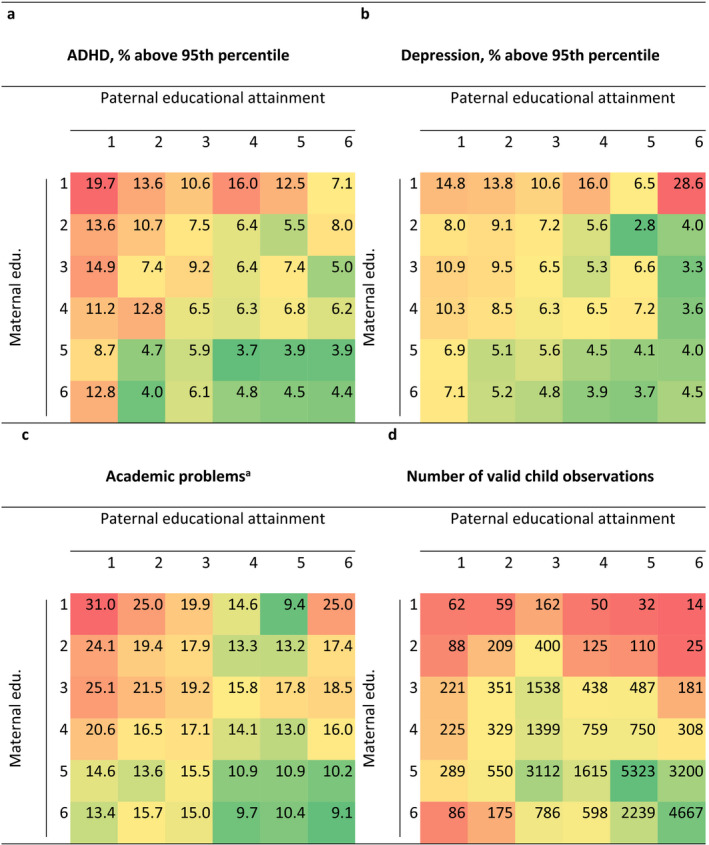
Proportion of children with high scores on (a) symptoms of attention deficit hyperactivity disorder (ADHD), (b) symptoms of depression, and (c) academic problems, and (d) the number of valid child observations by maternal and paternal educational attainment [Colour figure can be viewed at wileyonlinelibrary.com]

The cells are colored in shades from green to red to ease interpretation. The nuances reflect a gradient from low (green) to high (red) occurrence of problems or high (green) to low (red) numbers of valid observations. Educational attainment ranges from 1 (9‐year secondary school) to 6 (University, technical college, more than 4 years). $95\%\text{CI}=p\pm 1.96\sqrt{p\left(1-p\right)/n}]]>$.

^a^Includes children whose mothers reported ‘Teacher is concerned’ on at least one of the two items or ‘Must work more but teacher is not concerned’ on both items.

**Figure 2:**
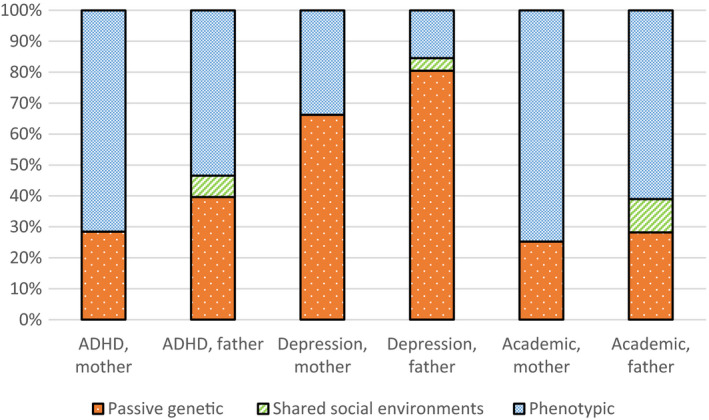
Sources of intergenerational correlation between parental educational attainment and symptoms of ADHD, symptoms of depression, and academic problems in children. The proportions are based on the full models. Phenotypic transmission was not present in the best fitting model for depression. Shared social environments were not present in any of the best fitting models [Colour figure can be viewed at wileyonlinelibrary.com]

Table 3 shows the results of the biometric model fitting and parameter estimates for each mode of intergenerational transmission under each model, including the full and best fitting models. Figure 2 shows the proportion of covariation explained by each mechanism in the full models for each outcome. The best fitting model for child symptoms of ADHD included paths directly from maternal and paternal educational attainment (model 7). Higher maternal and paternal educational attainment was associated with fewer symptoms of ADHD (−0.65 and −0.42 T‐points per level of education). A model which additionally included shared genetic effects had similar fit in terms of AIC (model 3). The full model for child symptoms of depression indicated that there may be a small phenotypic effect of maternal educational attainment. However, in the best fitting model, the intergenerational association between parental educational attainment and child symptoms of depression was fully explained by genetic factors related to both phenotypes and inherited from parents to children (model 5). In this model, there were no direct paths from parental education to depressive symptoms. In the best fitting model for academic problems, there were direct paths from maternal (−0.41 T‐points) and paternal (−0.41 T‐points) educational attainment to child academic problems (model 3). In addition, genetic factors that influenced parental education were associated with fewer academic problems in the child generation. The best fitting models are detailed in Figures S3–S5.

**Table 3:** Results of model fitting for the intergenerational association between maternal and paternal educational attainment and symptoms of attention deficit hyperactivity disorder (ADHD), symptoms of depression, and academic problems, including 95% confidence intervals

#	Model	Δ‐2LL	Δ*df*	ΔAIC	Shared genetic (A)	Shared environment (C)	mother (M)	Father (F)
Parental educational attainment and child symptoms of ADHD								
1	ACMF	–	–	–	−0.61 (−0.92, −0.29)	−0.13 (−0.29, 0.03)	−0.46 (−0.62, −0.29)	−0.20 (−0.31, −0.09)
2	–CMF	1.67	1	−0.32	0	−0.21 (−0.38, −0.05)	−0.71 (−0.81, −0.62)	−0.32 (−0.44, −0.20)
3	A–MF	0.15	1	−1.85	−0.65 (−1.03, −0.26)	0	−0.41 (−0.59, −0.23)	−0.25 (−0.39, −0.10)
4	AC––	4.81	2	0.81	−1.63 (−1.82, −1.45)	0.03 (−0.18, 0.24)	0	0
5	A–––	4.86	3	−1.14	−1.62 (−1.95, −1.29)	0	0	0
6	–C––	44.67	3	38.67	0	−0.94 (−1.07, −0.81)	0	0
**7**	––**MF**	**2.11**	**2**	−**1.89**	**0**	**0**	−**0.65 (−0.78, −0.53)**	−**0.42 (−0.52, −0.32)**
8	––––	371.51	4	363.51	0	0	0	0
Parental educational attainment and child symptoms of depression								
1	ACMF	–	–	–	−1.06 (−1.29, −0.83)	−0.06 (−0.27, 0.15)	−0.16 (−0.30, −0.02)	−0.05 (−0.17, 0.07)
2	–CMF	5.26	1	3.26	0	−0.19 (−0.30, −0.08)	−0.60 (−0.69, −0.51)	−0.28 (−0.38, −0.17)
3	A–MF	0.04	1	−1.96	−1.08 (−1.44, −0.71)	0	−0.14 (−0.30, 0.02)	−0.07 (−0.27, 0.12)
4	AC––	0.57	2	−3.43	−1.39 (−1.60, −1.18)	0.02 (−0.20, 0.23)	0	0
**5**	**A**–––	**0.58**	**3**	−**5.42**	−**1.39 (−1.60, −1.18)**	**0**	**0**	**0**
6	–C––	33.69	3	27.69	0	−0.83 (−0.96, −0.70)	0	0
7	––MF	5.62	2	−1.62	0	0	−0.55 (−0.68, −0.42)	−0.36 (−0.46, −0.27)
8	––––	272.13	4	264.13	0	0	0	0
Parental educational attainment and child academic problems								
1	ACMF	–	–	–	−0.59 (−0.87, −0.32)	−0.27 (−0.42, −0.11)	−0.53 (−0.64, −0.41)	−0.31 (−0.44, −0.18)
2	–CMF	1.51	1	−0.49	0	−0.38 (−0.58, −0.18)	−0.78 (−0.87, −0.70)	−0.42 (−0.58, −0.26)
**3**	**A**–**MF**	**0.55**	**1**	−**1.45**	−**0.69 (−0.96, −0.42)**	**0**	−**0.41 (−0.56, −0.27)**	−**0.41 (−0.53, −0.29)**
4	AC––	7.13	2	3.13	−1.86 (−2.04, −1.69)	−0.18 (−0.39, 0.02)	0	0
5	A–––	8.94	3	2.94	−1.93 (−2.30, −1.56)	0	0	0
6	–C––	58.47	3	52.47	0	−1.67 (−1.93, −1.41)	0	0
7	––MF	2.70	2	−1.30	0	0	−0.67 (−0.79, −0.56)	−0.59 (−0.70, −0.49)
8	––––	525.18	4	517.18	0	0	0	0

Best fitting models in bold. −2LL = −2 log likelihood; *df* = degrees of freedom; AIC = Akaike information criterion. A = effect of additive genetic factors for parental educational attainment on child outcomes; C = effect of shared environmental factors for parental educational attainment on child outcomes; M = effect of mother's educational attainment on child outcomes; F = effect of father's educational attainment and child outcomes. All outcomes are T‐scaled with a mean of 50 and standard deviation (*SD*) of 10.

Biometric model fitting with logarithmically transformed variables, presented in Table S5, led to the same conclusions regarding the presence or absence of phenotypic effects of maternal and paternal educational attainment.

## Discussion

Low educational attainment in parents was associated with symptoms of ADHD, symptoms of depression, and academic problems in children. After taking shared genetic and environmental factors into account, maternal educational attainment and paternal educational attainment were associated with child ADHD and academic problems, whereas the association with symptoms of depression could be fully explained by risk factors shared by the two generations.

### Symptoms of ADHD

The difference in risk of high levels of ADHD symptoms between the lowest and the highest educational groups demonstrates a strong educational gradient in ADHD. The association between parental educational attainment and symptoms of ADHD could not be fully ascribed to shared familial risk factors, and suggests effects of the parental phenotype. This result is in line with previous research that was not able to control for genetic predispositions (Russell, Ford, Rosenberg, & Kelly, 2014) and resonates well with a previous natural experiment on socioeconomic disadvantage and psychopathology (Costello et al., 2003). Previous research has demonstrated genetic correlations between adult education and child ADHD (de Zeeuw et al., 2014). Our finding of phenotypic influences is reconcilable with this, because phenotypic effects of parents on children are a potential source of genetic correlations. The parents' genotypes shape the environment of children they live with, and specifically, genetic liability to educational attainment has been found to influence parenting style (Wertz et al., 2019).

A model with shared genetic risk factors in addition to the phenotypic effects had almost as good fit to the data. This model may be more theoretically plausible: A parent's low educational attainment could in part reflect their own risk of ADHD (de Zeeuw, van Beijsterveldt, Ehli, de Geus, & Boomsma, 2017). In the degree to which ADHD is genetically influenced, one would expect children to inherit this genetic risk, and therefore to be at risk of ADHD partly due to passive genetic transmission. In any case, the presence of phenotypic effects in the two best fitting models indicates that genetic transmission of risk of ADHD does not fully explain the parent–child association. An alternative interpretation of the phenotypic effects is that environmental causes of parental ADHD influence parental education and are still present and influence ADHD in their children at age 8. This less parsimonious explanation cannot be ruled fully out and could be addressed in future studies.

#### Symptoms of depression

Parental education was associated with symptoms of depression, but not as strongly as with ADHD symptoms and primarily or exclusively through shared genetic factors. This does not mean that a causal effect cannot exist, but the full model suggests that it is small at best. The lack of phenotypic effects contrasts with studies on income (Akee et al., 2018; Zachrisson & Dearing, 2015). This discrepancy may be due to different effects of education versus income, or it may be that low education is not sufficiently disadvantageous to produce detectable influences on depression in present‐day Norway. Changes in income may be important for internalizing problems (Zachrisson & Dearing, 2015); however, educational attainment is primarily stable. Shared genetic factors for educational attainment and depression have been found before (Jansen et al., 2018). Our results demonstrate their relevance in explaining intergenerational associations.

#### Academic problems

Genetic transmission and remaining phenotypic associations each explained roughly half of the association between parental education and academic problems. Both mechanisms have been described in previous research (Black et al., 2005; Jansen et al., 2018; Kong et al., 2018). Our results indicate that parental genetic factors are associated with outcomes in children partly because they shape the children's environment. Lagging academically from an early age may influence children's academic careers, with consequences for their mental health and educational attainment in adulthood.

#### Implications

We cannot prove that parental educational attainment causally influences ADHD or academic problems, but this possibility is strengthened by the failure to falsify the causal hypotheses. If substantiated, these findings imply that interventions enhancing parental education could improve mental health and academic success in children at risk. There are several possible benefits from high educational attainment in the parents. Parental education may lead to improved living conditions, increased understanding of children's needs, or beneficial parental practices. These mediators may provide additional targets for preventive interventions. Further studies are needed to understand exactly how parental education may have beneficial effects on children's development. We observed high relative risks between the highest and lowest educational categories that were comparable to previous studies (Hjern et al., 2010; Russell et al., 2016). Nevertheless, parental education explained only a small proportion of the variance in the child outcomes (≈1%). Identifying and intervening on mediators that exist in all educational groups, but are more common among groups with low educational attainment, may have a higher preventive potential than intervening directly on educational level. Intervening directly on the educational attainment of a population could yield positive results if there are effects of absolute educational level. If, however, there are effects of the relative rank, benefits of increasing some group's level of education could come at the cost of other groups. In populations similar to this, interventions aimed at parental education are likely to have no or only small effects on the children's depressive symptoms.

The similarity between partners in genetic factors for educational attainment was high (*r* = .73) and comparable to estimates derived from genomic data (.65) (Robinson et al., 2017). Genetic similarity between partners can increase the genetic variation in following generations, with possible consequences for social inequalities and the prevalence of mental disorders (Peyrot, Robinson, Penninx, & Wray, 2016). The processes leading to the high genetic spouse correlations in educational attainment are as yet unknown, and a better understanding of these processes may be useful for understanding the etiology mental disorders.

#### Limitations

Despite strengths such as a population‐based sample and a quasi‐experimental quantitative genetic design that accounts for all genetic variance, this study has some limitations: First, it could bias our results if mothers with low educational attainment have a lower threshold for reporting symptoms or report academic problems less precisely. However, the associations were approximately equally strong for fathers, who did not report on their children. Second, parental educational attainment may not capture the socioeconomic factors relevant for depressive symptoms. We focused on education because this has been the target of genetically informative studies. Third, parents with high educational attainment were more likely to participate, which may compromise generalizability of our findings. Nevertheless, association estimates from MoBa regarding ADHD have been found to have reasonable generalizability to the general population (Oerbeck et al., 2017). Similar analyses are not available for the other two studied outcomes. Our use of educational data on parents who later dropped out is likely to reduce participation bias. Fourth, the generalizability may be constrained to societies with similar distribution of educational opportunities. Fifth, like all null findings, the absence of significant effects of parental education on child depression does not prove that such effects do not exist. Sixth, limited power or violations of the equal environments assumption could lead to mixing of shared environment and additive genetic effects. However, parent–child associations adjusted for both these factors would be unaffected by such biases.

## Conclusions

Symptoms of ADHD, symptoms of depression, and academic problems in 8‐year‐old children were more common among children of mothers and fathers with low educational attainment. Shared genetic risk partially accounted for the association with depression and academic problems, and possibly also for child ADHD. For symptoms of ADHD and academic problems, but not symptoms of depression, the associations remained after accounting for genetic and environmental risk factors shared in families. Thus, factors related to parental education might be targets of interventions aimed at reducing symptoms of ADHD and academic problems, whereas parental education primarily indicates genetic risk for symptoms of depression.

## Supporting information


**Table S1** **.** Number of complete sibling pairs in the parent generation and discordance in educational attainment by kinship type.
**Table S2** **.** Bivariate correlations between parental educational attainment and offspring outcomes, including 95% confidence intervals.
**Table S3** **.** Results of linear regression of symptoms of attention deficit hyperactivity disorder (ADHD), symptoms of depression, and academic problems on parental educational attainment, child sex, parental age, and year of birth.
**Table S4** **.** Results of binary logistic regression of high levels of symptoms of attention deficit hyperactivity disorder (ADHD), depression, and academic problems by parental educational attainment, child sex, parental age, and year of birth.
**Table S5** **.** Results of model fitting for the intergenerational association between maternal and paternal educational attainment and logarithmically transformed outcome variables in children, including 95% confidence intervals.
**Figure S1** **.** The full biometric model.
**Figure S2** **.** Distribution of the outcome variables before and after logarithmic Transformation.
**Figure S3** **.** Best fitting model for parental educational attainment and child symptoms of attention deficit hyperactivity disorder (ADHD).
**Figure S4** **.** Best fitting model for parental educational attainment and child symptoms of depression.
**Figure S5** **.** Best fitting model for parental educational attainment and child academic problems.
