# Supplementary material for: Mechanisms linking parental educational attainment with child ADHD, depression, and academic problems: a study of extended families in The Norwegian Mother, Father and Child Cohort Study
Source: J Child Psychol Psychiatry. 2020 Jan 19;61(9):1009–18. doi: 10.1111/jcpp.13197 (PMC8607471; doi:10.1111/jcpp.13197)
Supplement: Supplementary file 1 — Table S1 . Number of complete sibling pairs in the parent generation and discordance in educational attainment by kinship type. Table S2 . Bivariate correlations between parental educational attainment and offspring outcomes, including 95% confidence intervals. Table S3 . Results of linear regression of symptoms of attention deficit hyperactivity disorder (ADHD), symptoms of depression, and academic problems on parental educational attainment, child sex, parental age, and year of birth. Table S4 . Results of binary logistic regression of high levels of symptoms of attention deficit hyperactivity disorder (ADHD), depression, and academic problems by parental educational attainment, child sex, parental age, and year of birth. Table S5 . Results of model fitting for the intergenerational association between maternal and paternal educational attainment and logarithmically transformed outcome variables in children, including 95% confidence intervals. Figure S1 . The full biometric model. Figure S2 . Distribution of the outcome variables before and after logarithmic Transformation. Figure S3 . Best fitting model for parental educational attainment and child symptoms of attention deficit hyperactivity disorder (ADHD). Figure S4 . Best fitting model for parental educational attainment and child symptoms of depression. Figure S5 . Best fitting model for parental educational attainment and child academic problems. [file JCPP-61-1009-s001.docx]

SUPPORTING INFORMATION

**MECHANISMS LINKING PARENTAL EDUCATIONAL ATTAINMENT WITH CHILD ADHD, DEPRESSION, AND ACADEMIC PROBLEMS: A STUDY OF EXTENDED FAMILIES IN THE NORWEGIAN MOTHER, FATHER, AND CHILD COHORT STUDY**

Fartein Ask Torvik, Espen Moen Eilertsen, Tom A. McAdams, Kristin Gustavson, Henrik Daae Zachrisson, Ragnhild Brandlistuen, Line C. Gjerde, Alexandra Havdahl, Camilla Stoltenberg, Helga Ask, Eivind Ystrom

**TABLE OF CONTENTS** page

Table S1. Number of complete sibling pairs in the parent generation and

discordance in educational attainment by kinship type 2

Table S2. Bivariate correlations between parental educational attainment and

offspring outcomes, including 95% confidence intervals 3

Table S3. Results of linear regression of symptoms of attention deficit hyperactivity

disorder (ADHD), symptoms of depression, and academic problems on

parental educational attainment, child sex, parental age, and year of birth 4

Table S4. Results of binary logistic regression of high levels of symptoms of

attention deficit hyperactivity disorder (ADHD), depression, and academic

problems by parental educational attainment, child sex, parental age, and

year of birth 5

Table S5. Results of model fitting for the intergenerational association between

maternal and paternal educational attainment and logarithmically

transformed outcome variables in children, including 95% confidence intervals 6

Figure S1. The full biometric model 7

Figure S2. Distribution of the outcome variables before and after logarithmic

transformation 9

Figure S3. Best fitting model for parental educational attainment and child symptoms

of attention deficit hyperactivity disorder (ADHD) 10

Figure S4. Best fitting model for parental educational attainment and child

symptoms of depression 11

Figure S5. Best fitting model for parental educational attainment and child

academic problems 12

**Table S1. Number of complete sibling pairs in the parent generation and discordance in educational attainment by kinship type.**

| Type of relationship | Paternal half-siblings | Maternal half-siblings | Full siblings | Dizygotic twins | Monozygotic twins |
| --- | --- | --- | --- | --- | --- |
| Number of complete parental sibling pairs | 307 | 271 | 8147 | 78 | 69 |
| Number discordant | 234 | 194 | 2893 | 58 | 27 |
| Proportion discordant | 76.22% | 71.59% | 35.51% | 74.36% | 39.1% |
| Average difference | 1.50 | 1.23 | 0.99 | 1.06 | 0.57 |
| Average difference for discordant couples | 1.97 | 1.71 | 1.54 | 1.43 | 1.46 |
| Sibling correlation (95% CI) | 0.11  (0.00, 0.22) | 0.31  (0.20, 0.42) | 0.42  (0.40, 0.44) | 0.39  (0.19, 0.57) | 0.50  (0.30, 0.66) |

Notes: Parents participating without siblings are not counted. Average difference was measures with level of education scored 1 to 6 as the unit and calculated as the mean of absolute differences between siblings.

**Table S2. Bivariate correlations between parental educational attainment and offspring outcomes, including 95% confidence intervals.**

|  | Mother | Father |
| --- | --- | --- |
| Symptoms of ADHD | -0.08 (-0.09, -0.07) | -0.08 (-0.09, -0.07) |
| Symptoms of depression | -0.07 (-0.08, -0.06) | -0.07 (-0.08, -0.06) |
| Academic problems | -0.11 (-0.12, -0.10) | -0.11 (-0.12, -0.10) |

Note: Observed correlations and confidence intervals were identical for mothers and fathers when rounded to two digits.

**Table S3. Results of linear regression of symptoms of attention deficit hyperactivity disorder (ADHD), symptoms of depression, and academic problems on parental educational attainment, child sex, parental age, and year of birth.**

|  | **ADHD** | | **Depression** | | **Academic problems** | |
| --- | --- | --- | --- | --- | --- | --- |
|  | b | (95% CI) | b | (95% CI) | b | (95% CI) |
| **Model 1** |  | *(r^2^=0.0091)* |  | *(r^2^=0.0070)* |  | *(r^2^=0.0169)* |
| Education, mother | -0.48 | (-0.59, -0.37) | -0.43 | (-0.54, -0.32) | -0.65 | (-0.76, -0.53) |
| Education, father | -0.41 | (-0.49, -0.32) | -0.35 | (-0.44, -0.26) | -0.57 | (-0.66, -0.48) |
|  |  |  |  |  |  |  |
| **Model 2** |  | *(r^2^=0.0399)* |  | *(r^2^=0.0076)* |  | *(r^2^=0.0186)* |
| Sex (female), child | -3.50 | (-3.72, -3.28) | -0.47 | (-0.69, -0.25) | -0.80 | (-1.03, -0.58) |
| Education, mother | -0.48 | (-0.59, -0.37) | -0.43 | (-0.54, -0.32) | -0.65 | (-0.76, -0.53) |
| Education, father | -0.41 | (-0.50, -0.32) | -0.35 | (-0.44, -0.26) | -0.57 | (-0.66, -0.48) |
|  |  |  |  |  |  |  |
| **Model 3** |  | *(r^2^=0.0416)* |  | *(r^2^=0.0140)* |  | *(r^2^=0.0204)* |
| Sex (female), child | -3.49 | (-3.71, -3.27) | -0.45 | (-0.67, -0.23) | -0.82 | (-1.05, -0.60) |
| Education, mother | -0.45 | (-0.56, 0.34) | -0.36 | (-0.47, -0.25) | -0.63 | (-0.75, -0.52) |
| Education, father | -0.39 | (-0.48, -0.31) | -0.33 | (-0.42, -0.24) | -0.56 | (-0.65, -0.47) |
| Age, mother | -0.10 | (-0.13, -0.06) | -0.16 | (-0.20, -0.13) | 0.03 | (0.00, 0.07) |
| Age, father | 0.03 | (0.00, 0.06) | 0.00 | (-0.03, 0.03) | 0.01 | (-0.02, 0.04) |
| Year of birth | 0.14 | (0.07, 0.20) | 0.24 | (0.18, 0.31) | -0.22 | (-0.28, -0.15) |

Notes: b = regression coefficient. CI = confidence interval. Educational attainment is measured in levels (1-6).

**Table S4. Results of binary logistic regression of high levels of symptoms of attention deficit hyperactivity disorder (ADHD), depression, and academic problems by parental educational attainment, child sex, parental age, and year of birth.**

|  | **ADHD** | | **Depression** | | **Academic problems** | |
| --- | --- | --- | --- | --- | --- | --- |
|  | OR | (95% CI) | OR | (95% CI) | OR | (95% CI) |
| Sex (female), child | 0.41 | (0.37, 0.45) | 0.81 | (0.73, 0.90) | 0.89 | (0.83, 0.95) |
| **Maternal education** |  |  |  |  |  |  |
| 1. Lower secondary school (9 years) | 2.10 | (1.50, 2.94) | 2.37 | (1.68, 3.33) | 1.78 | (1.35, 2.33) |
| 2. Upper secondary, basic (10.5 years) | 1.43 | (1.09, 1.87) | 1.32 | (0.99, 1.76) | 1.44 | (1.19, 1.75) |
| 3. Vocational (11 years) | 1.41 | (1.18, 1.70) | 1.28 | (1.05, 1.55) | 1.67 | (1.47, 1.90) |
| 4. Upper secondary, completed (12 years) | 1.30 | (1.09, 1.55) | 1.34 | (1.12, 1.61) | 1.38 | (1.21, 1.56) |
| 5. University, short (15 years) | 0.85 | (0.74, 0.97) | 0.99 | (0.86, 1.13) | 1.09 | (1.00, 1.20) |
| 6. University, long (17 years) | 1.00 |  | 1.00 |  | 1.00 |  |
| **Paternal education** |  |  |  |  |  |  |
| 1. Lower secondary school (9 years) | 2.40 | (1.89, 3.05) | 1.79 | (1.38, 2.31) | 1.76 | (1.46, 2.12) |
| 2. Upper secondary, basic (10.5 years) | 1.51 | (1.20, 1.89) | 1.47 | (1.17, 1.84) | 1.49 | (1.27, 1.74) |
| 3. Vocational (11 years) | 1.35 | (1.15, 1.58) | 1.18 | (1.00, 1.38) | 1.50 | (1.35, 1.67) |
| 4. Upper secondary, completed (12 years) | 1.03 | (0.85, 1.25) | 1.02 | (0.84, 1.24) | 1.08 | (0.95, 1.23) |
| 5. University, short (15 years) | 1.04 | (0.89, 1.21) | 0.98 | (0.84, 1.14) | 1.08 | (0.98, 1.20) |
| 6. University, long (17 years) | 1.00 |  | 1.00 |  | 1.00 |  |
| Age, mother | 0.97 | (0.95, 0.98) | 0.96 | (0.95, 0.98) | 1.01 | (1.00, 1.02) |
| Age, father | 1.01 | (1.00, 1.02) | 1.00 | (0.99, 1.01) | 1.00 | (1.00, 1.01) |
| Year of birth | 1.03 | (1.00, 1.05) | 1.06 | (1.03, 1.09) | 0.95 | (0.94, 0.97) |

Notes: OR = odds ratio. CI = confidence interval. Symptoms of ADHD and symptoms of depression are dichotomized at the 95^th^ percentile. Academic problems include children whose mothers reported ‘Teacher is concerned’ on at least one of the two items or ‘Must work more but teacher is not concerned’ on both items. OR for combinations of groups is obtained by multiplication, for instance when both the mother and the father have short education (category 1), the OR for high levels of ADHD is 2.10 x 2.40 = 5.04, for high levels of depression 2.37 x 1.79 = 4.24, and for academic problems 1.78 x 1.76 = 3.13, compare to when both parents have long education (category 6). Adding interaction effects reduced the fit for all models.

**Table S5. Results of model fitting for the intergenerational association between maternal and paternal educational attainment and logarithmically transformed outcome variables in children, including 95% confidence intervals.**

| **Parental educational attainment and child symptoms of ADHD** | | | | | | | | |
| --- | --- | --- | --- | --- | --- | --- | --- | --- |
| # | Model | Δ-2LL | Δdf | ΔAIC | Shared genetic (A) | Shared environment (C) | Mother (M) | Father (F) |
| 1 | ACMF | - | - | - | -0.60 (-0.91, -0.29) | -0.18 (-0.45, 0.09) | -0.37 (-0.51, -0.23) | -0.18 (-0.39, 0.03) |
| 2 | –CMF | 1.31 | 1 | -0.69 | 0 | -0.28 (-0.61, 0.05) | -0.62 (-0.77, 0.47) | -0.22 (-0.44, 0.00) |
| **3** | **A–MF** | **0.58** | **1** | **-1.42** | **-0.73 (-1.69, 0.22)** | **0** | **-0.30 (-0.71, 0.11)** | **-0.27 (-0.47, -0.07)** |
| 4 | AC–– | 3.41 | 2 | -0.59 | -1.47 (-1.69, -1.25) | -0.21 (-0.38, -0.04) | 0 | 0 |
| 5 | A––– | 6.17 | 3 | 0.17 | -1.61 (-1.83, -1.38) | 0 | 0 | 0 |
| 6 | –C–– | 44.69 | 3 | 38.69 | 0 | -1.01 (-1.17, -0.85) | 0 | 0 |
| 7 | ––MF | 2.84 | 2 | -1.16 | 0 | 0 | -0.60 (-0.72, -0.48) | -0.39 (-0.49, -0.30) |
| 8 | –––– | 332.85 | 4 | 324.85 | 0 | 0 | 0 | 0 |
| **Parental educational attainment and child symptoms of depression** | | | | | | | | |
| # | Model | Δ-2LL | Δdf | ΔAIC | Shared genetic (A) | Shared environment (C) | Mother (M) | Father (F) |
| 1 | ACMF | - | - | - | -0.94 (-1.17, -0.70) | -0.20 (-0.41, 0.01) | -0.16 (-0.29, -0.02) | -0.09 (-0.27, 0.09) |
| 2 | –CMF | 3.40 | 1 | 1.40 | 0 | -0.32 (-0.51, -0.13) | -0.55 (-0.66, -0.43) | -0.17 (-0.30, -0.03) |
| 3 | A–MF | 0.76 | 1 | -1.24 | -1.06 (-1.49, -0.62) | 0 | -0.09 (-0.29, 0.10) | -0.19 (-0.31, -0.07) |
| **4** | **AC––** | **0.70** | **2** | **-3.30** | **-1.31 (-1.54, -1.08)** | **-0.23 (-0.40, -0.06)** | **0** | **0** |
| 5 | A––– | 4.23 | 3 | -1.77 | -1.47 (-1.67, -1.27) | 0 | **0** | **0** |
| 6 | –C–– | 37.04 | 3 | 31.04 | 0 | -0.90 (-1.05, -0.76) | 0 | 0 |
| 7 | ––MF | 5.55 | 2 | 1.55 | 0 | 0 | -0.53 (-0.64, -0.42) | -0.37 (-0.46, -0.28) |
| 8 | –––– | 269.89 | 4 | 261.89 | 0 | 0 | 0 | 0 |
| **Parental educational attainment and child academic problems** | | | | | | | | |
| # | Model | Δ-2LL | Δdf | ΔAIC | Shared genetic (A) | Shared environment (C) | Mother (M) | Father (F) |
| 1 | ACMF | - | - | - | -0.55 (-0.92, -0.17) | -0.19 (-0.49, 0.10) | -0.47 (-0.62, -0.32) | -0.39 (-0.62, -0.16) |
| 2 | –CMF | 0.92 | 1 | -1.08 | 0 | -0.30 (-0.68, 0.08) | -0.70 (-0.88, -0.52) | -0.41 (-0.59, -0.22) |
| **3** | **A–MF** | **0.51** | **1** | **-1.49** | **-0.72 (-1.73, 0.28)** | **0** | **-0.38 (-0.82, 0.05)** | **-0.48 (-0.68, -0.27)** |
| 4 | AC–– | 8.28 | 2 | 4.28 | -1.75 (-2.04, -1.47) | -0.40 (-0.64, -0.16) | 0 | 0 |
| 5 | A––– | 15.32 | 3 | 9.32 | -1.96 (-2.20, -1.71) | 0 | 0 | 0 |
| 6 | –C–– | 57.55 | 3 | 51.55 | 0 | -1.50 (-1.78, -1.22) | 0 | 0 |
| **7** | **––MF** | **2.48** | **2** | **-1.52** | **0** | **0** | **-0.68 (-0.81, -0.55)** | **-0.60 (-0.72, -0.49)** |
| 8 | –––– | 538.11 | 4 | 530.11 | 0 | 0 | 0 | 0 |

Notes: Best fitting models in bold. -2LL = -2 log likelihood; df = degrees of freedom; AIC = Akaike Information Criterion. A = effect of additive genetic factors for parental educational attainment on child outcomes; C = effect of shared environmental factors for parental educational attainment on child outcomes; M = effect of mother’s educational attainment on child outcomes; F = effect of father’s educational attainment and child outcomes. The outcome variable are logarithmically transformed to reduce skewness. The outcomes are T-scaled with a mean of 50 and standard deviation (SD) of 10 after the logarithmic transformation to enhance comparison with main results.

**Figure S1. The full biometric model.** A = additive genetic factors; C = shared (family) environmental factor; E = individual-specific environmental factors. Parameters shown in green were freely estimated.

The following parameters are relevant in the parent generation: We allowed the relative importance of A, C, and E influences on educational attainment to be different for mothers (a_m_, c_m_, e_m_) and fathers (a_f_, c_f_, e_f_). Correlations between mothers and fathers in A, C, and E factors are freely estimated as r_Ap_, r_Cp_, and r_Ep_, respectively. r_G_ refer to the coefficient of relatedness. In order to account for genetic resemble between partners (r_Ap_ > 0), we set r_G_ to 1 for monozygotic (MZ) twins, $1/2+{(1}/2)r_{Ap}$ for dizygotic (DZ) twins and full siblings, and $1/4+{(1}/{2)}r_{Ap}+{(1}/{4)}{r_{Ap}}^{2}$ for maternal and paternal half-siblings. If there is no genetic resemblance between partners (r_Ap_ = 0) these numbers reduce to the typical values of 1 for MZs, $1/2$ for DZs and full siblings, and $1/4$ for maternal and paternal half-siblings. r_C_ is defined as the resemblance in the environment that siblings share and was set to 1 for all sibling types except paternal half-siblings, for which it was set to 0. In-law and co-in-law correlations in A, C, and E are defined as the product of sibling and partner correlations in these factors.

The following parameters are relevant for the associations between parents and offspring: We refer to passive genetic transmission as a_c1_. This is the effect of genetic influences on parental educational attainment on the offspring outcome. Likewise, we refer to passive shared environmental transmission as c_c_, which is the effect that shared environmental influences on parental educational attainment have on the offspring outcome. b_cm_ is the effect of maternal educational attainment on the offspring outcome after accounting for shared risk factors in the two generations (a_c1_ and c_c_) and b_cf_ is the effect of paternal educational attainment on the offspring outcome after accounting for the shared risk factors. If a factor influences the education of an individual, but not his or her sibling’s education, and this factor is associated with an outcome in the individual’s offspring, but not in the sibling’s offspring, that could indicate effects of the educational attainment on the offspring outcome. Such factors could include stochastic events, which are included in E, and genetic factors that were not transmitted to the next generation, which are included in A, but not in A1.

Residual variation in offspring outcomes that was independent of parental educational attainment was decomposed into separate A’, C’, and E’ components. In the child generation, the genetic resemblance between siblings is 1/2, as only full siblings were included, and the shared environment is correlated 1 between siblings. Between cousins, r_Gchild_ is fixed to ¼ for offspring of MZ twins, 1/8 for offspring of full siblings or DZ twins, and 1/16 for offspring of maternal or paternal half-siblings. C’ in the offspring generation can be substituted with dominant genetic effects (D’), which are correlated ¼ between full siblings, and 0 between cousins.

All latent variables were specified to have unit variance. Report refers to parental educational attainment assessed in questionnaires during pregnancy for child 1 and child 2, which were combined in a measurement model with the loadings α_m_ for mothers and α_f_ for fathers.


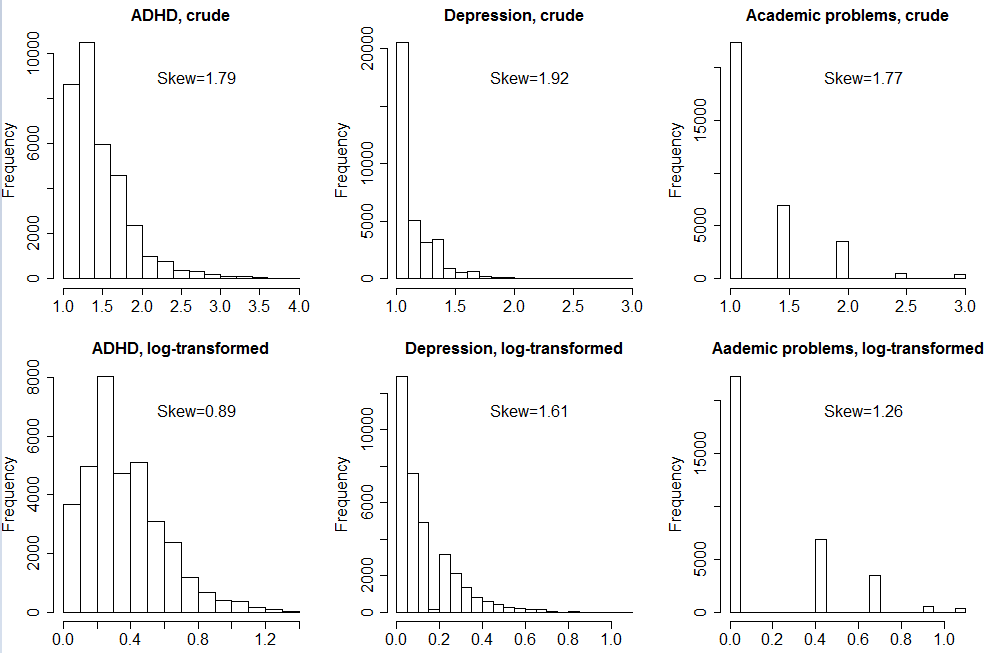


**Figure S2. Distribution of the outcome variables before (upper) and after (lower) logarithmic transformation.**

**Figure S3. Best fitting model for parental educational attainment and child symptoms of attention deficit hyperactivity disorder (ADHD).** Edu_m_ = maternal educational attainment. Edu_f_ = paternal educational attainment. Educational attainment is scaled from 1 (secondary school) to 6 (university, long), and symptoms of ADHD are T-scaled (mean of 50 and standard deviation of 10). The difference between the highest and lowest educational group is therefore five times as large as the numbers presented here. A = additive genetic factors, C = shared environmental factors, D = dominant genetic factors, E = non-shared or individual-specific environment. A model with D in the child generation had better fit than a model with C (Δ*-2LL* = -0.15, Δ*df* = 0). We therefore included D in the child generation.

**Figure S4. Best fitting model for parental educational attainment and child symptoms of depression.** Edu_m_ = maternal educational attainment. Edu_f_ = paternal educational attainment. Educational attainment is scaled from 1 (secondary school) to 6 (university, long), and symptoms of depression are T-scaled (mean of 50 and standard deviation of 10). The difference between the highest and lowest educational group is therefore five times as large as the numbers presented here. A = additive genetic factors, C = shared environmental factors, E = non-shared or individual-specific environment. A model with dominant genetic factors in the child generation had worse fit than a model with C (Δ*-2LL* = 15.24, Δ*df* = 0). We therefore included C in the child generation.

**Figure S5. Best fitting model for parental educational attainment and child academic problems.** Edu_m_ = maternal educational attainment. Edu_f_ = paternal educational attainment. Educational attainment is scaled from 1 (secondary school) to 6 (university, long), and academic problems are T-scaled (mean of 50 and standard deviation of 10). The difference between the highest and lowest educational group is therefore five times as large as the numbers presented here. A = additive genetic factors, C = shared environmental factors, D = dominant genetic factors, E = non-shared or individual-specific environment. A model with D in the child generation had better fit than a model with C (Δ*-2LL* = -30.55, Δ*df* = 0). We therefore included D in the child generation.
